# An Overmoded-Waveguide-Based Permittivity Measurement Method with High Accuracy and Ultra-Broadband over 8–110 GHz

**DOI:** 10.3390/mi16091045

**Published:** 2025-09-12

**Authors:** Weijie Wang, Yingjian Cao, Tieyang Wang, Fangfang Song, Shuanzhu Fang, Xianfeng Tang, Xiangqiang Li, Guoxiang Shu, Guo Liu

**Affiliations:** 1 School of Physical Science and Technology, Southwest Jiaotong University, Chengdu 611756, China; wangweijie@swjtu.edu.cn (W.W.); txf_2012@163.com (X.T.); xiangqiang_li@swjtu.edu.cn (X.L.); 2 School of Electronic Science and Engineering, University of Electronic Science and Technology of China, Chengdu 610054, China; cyj18883196024@163.com (Y.C.); tieyangwang@foxmail.com (T.W.); 3 China Electronic Production Reliability and Environmental Testing Research Institute, Guangzhou 511375, China; songff@ceprei.com (F.S.); fangshuanzhu@163.com (S.F.); 4 College of Electronics and Information Engineering, Shenzhen University, Shenzhen 518060, China; gxshu@szu.edu.cn

**Keywords:** permittivity measurement, dielectric material, broadband, microwave, sub-terahertz

## Abstract

An overmoded-waveguide-based kit operating in 8–110 GHz for material complex permittivity measurement is proposed and designed in this paper. It overcomes the significant errors caused by air gaps in the conventional standard waveguide method (SWM), especially for millimeter-wave frequency bands. Furthermore, it avoids the problem of SWM requiring different samples in broadband measurements. The proposed kit consists of an overmoded-waveguide sample fixture with cross dimensions of 22.86 mm × 10.16 mm, seven pairs of standard-overmoded waveguide transition structures for different frequency bands, and thru-reflect-line calibration kits. The air gap problem, a major error source in millimeter-wave measurement, is quantitatively investigated. Compared with the SWM method, the proposed kit can decrease errors from over 68% to below 8%. The proposed method was verified by measuring the polytetrafluoroethylene sample. Then, it was applied to measure the BeO-TiO_2_ ceramic, which is widely used in vacuum devices. The measured data are valuable for applying BeO-TiO_2_ ceramics in relevant devices and developing its dielectric relaxation model.

## 1. Introduction

Dielectric materials are widely used in microwave and millimeter-wave techniques, such as wireless communications, energy harvesting, and vacuum devices [1,2,3]. In these applications, accurate dielectric permittivity measurement is essential. Many methods are available for material permittivity measurement and can be divided into resonance-based and transmission/reflect (T/R) methods [4]. The resonance method is mainly used for high-precision measurement of ultra-low loss materials at single or several discrete frequencies. In contrast, the T/R method is mainly used in broadband measurement for medium/high loss materials. Among the T/R methods, the waveguide method is widely applied in microwave bands due to its simplicity, high accuracy, convenience, and appropriate sample size.

However, the conventional standard waveguide method (SWM) faces two challenges as measurement requirements expand to millimeter-wave bands. On the one hand, the air gap problem can cause significant errors as the frequency increases to millimeter-wave bands [5,6,7,8]. On the other hand, due to the different sizes of sample fixtures used in each frequency band, the samples have to be replaced in the measurement at different frequency bands. When the consistency of individual samples cannot be guaranteed, the differences between samples can lead to inaccurate broadband properties. This will mislead the study of the dielectric mechanism and the prediction of the out-of-band properties of the material. Therefore, it is necessary to improve the conventional waveguide method to achieve a high accuracy and ultra-broadband measurement (for a same sample) in microwave to millimeter-wave bands.

In order to solve the air gap problem of the waveguide method, researchers have proposed several error correction models and algorithms, such as the layered capacitor model, modal matching analysis, β factor correction algorithm, and numerical methods [8,9,10,11]. Other efforts focused on the improvement of measurement devices. Ref. [12] presented a fixture with a gap-filling structure. Compared to the conventional waveguide method, it can reduce the errors from 10% to 2% at the Ka band. Refs. [13,14] proposed an overmoded waveguide method (OWM) to enhance air gap tolerance. Compared to the conventional waveguide method, it extends the dimensions of the standard waveguide and uses the overmoded rectangular TE_10_ or TE_01_ mode as the operating mode. This method can achieve a measurement error below 5% at the W band. Ref. [15] proposed a waveguide-clamped method. This method can accurately (uncertainty below 5% at V-band) and quickly measure thin films below 1/10 wavelength. Alternatively, Ref. [16] adopted a circular waveguide operating with TE_01_ mode in permittivity measurements. This method utilizes the fact that the field of the circular TE_01_ mode is not distributed on the waveguide wall, making the measurement results insensitive to the air gap. These works greatly extend the applications of the waveguide method.

Despite the improvement in measurement accuracy, there is a lack of research on broadband measurement (multi-octave bandwidth) for the same sample. As mentioned above, since the waveguide method is limited by the cutoff frequency, different sizes of samples and fixtures are required in different frequency band measurements. However, with the development of microwave and millimeter-wave techniques, ultra-broadband measurement of dielectric permittivity is becoming an increasingly important requirement. This not only directly affects the design of ultra-wideband devices, but also provides an important basis for the study of material dielectric mechanisms and the development of new materials. To address these two problems, we proposed an OWM-based ultra-broadband measurement method in this paper. Since the overmoded waveguide significantly decreases the cutoff frequency of the operation mode, this method allows for measuring the same sample in 8–110 GHz. At the same time, the use of an overmoded waveguide will greatly decrease the measurement error caused by the air gap.

This article is organized as follows. The design of the OWM-based ultra-broadband kit is presented in Section 2. Then, a quantitative error analysis of the proposed kit will be performed and compared with the SWM. Section 3 presents the measurement results of the proposed kit. A polytetrafluoroethylene (PTFE) sample is measured in 8–110 GHz, and then the measured data are compared to previously published data. Furthermore, the proposed kit is applied in the measurement of the BeO-TiO_2_ ceramic. Finally, a discussion and conclusion are provided in Section 4.

## 2. Design of the OWM-Based Measurement Kit

**Figure 1:**
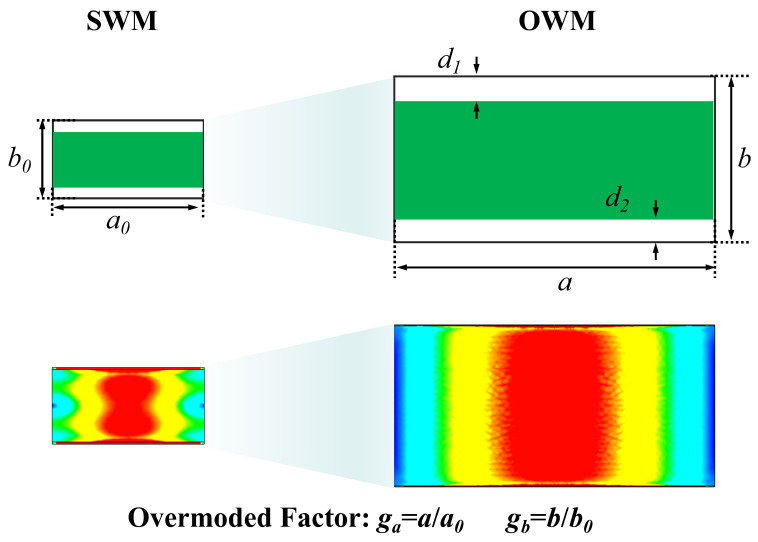
Electric field diagrams of the loaded fixture by SWM and OWM with the air gap.

To overcome the air gap problem and realize ultra-broadband measurement for the same sample, we adopt the OWM-based measurement technique in this paper. Figure 1 shows the fixtures of the loaded sample with E-plane air gaps *d*_1_ and *d*_2_ and the electric field distribution of the operating modes in SWM and OWM. In the OWM, the *a*_0_ and *b*_0_ sides of the standard waveguide are expanded to *a* and *b*. Thus, we can define the overmoded factor as $g_{a}$ = *a*/$a_{0}$ and $g_{b}$ = *b*/$b_{0}$. Since the relative size of the air gap is reduced as *g* increases, its effect will be significantly alleviated. Figure 1 presents the electric field of the operating mode in the SWM (standard waveguide at the Ka band with dimensions of 7.12×3.56mm) and in the OWM with a overmoded factor *g* of 5 at 33 GHz. Here, the considered material has $\epsilon_{r}^{\prime }$ and tanδ of 15 and 0.5, respectively. The gap size $d_{1}$ and $d_{2}$ are 0.05 mm. The operating mode (distorted $TE_{10}$ mode) in material-loaded waveguides are excited by a hollow waveguide $TE_{10}$ mode. As depicted in Figure 1, the operating rectangular ${\mathrm{TE}}_{10}$ mode has severe distortion in the SWM, whereas the mode distortion in the OWM is slight.

To illustrate the effect of air gaps on measurement accuracy in SWM, we analyze three typical frequency bands: X (microwave), Ka (millimeter-wave), and W (sub-terahertz) bands. In order to quantitatively study the error caused by the air gap, we simulate the waveguide model of the loaded sample under the air gap by CST MW Studio [17] and obtain the corresponding S-parameters. Then, the complex permittivity is extracted by solving the equation proposed in Ref. [18], which assumes the sample is completely filled in the waveguide:

(1) $$\frac{1}{2}\left\{\left[S_{12}+S_{21}\right]+\beta \left[S_{11}+S_{22}\right]\right\}=\frac{z\left(1-\Gamma^{2}\right)+\beta \Gamma \left(1-z^{2}\right)}{1-z^{2}\Gamma^{2}}$$

Here, S-parameters are the measured data. Variables *z* and Γ represent the transmission and reflection coefficients of the sample-loaded waveguide, respectively, which are functions of the complex permittivity $\epsilon_{r}$ and permeability $\mu_{r}$ of the material under test. For non-magnetic materials with $\mu_{r}=1$, the complex permittivity can be determined by solving Equation (1) numerically. Since unavoidable air gaps can cause deviations in the measured S-parameters and ultimately lead to errors in the extracted permittivity in practice, it is necessary to perform a quantitative error analysis.

**Figure 2:**
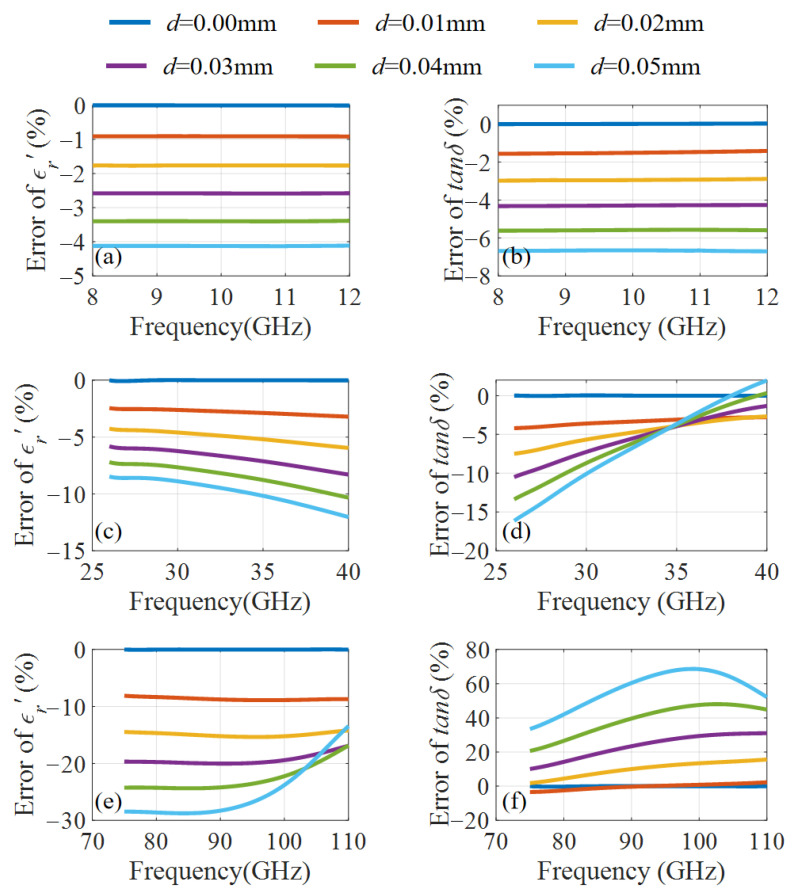
Measurement errors of $\epsilon_{r}^{\prime }$ and tanδ in various frequency bands with different air gaps *d*. (**a**) errors of $\epsilon_{r}^{\prime }$ at X–band, (**b**) errors of tanδ at X–band, (**c**) errors of $\epsilon_{r}^{\prime }$ at Ka–band, (**d**) errors of tanδ at Ka–band, (**e**) errors of $\epsilon_{r}^{\prime }$ at W–band, (**f**) errors of tanδ at W–band.

To simplify the analysis while maintaining its generality, we assume that the air gaps at the top and bottom ends are the same in simulation, i.e., $d_{1}$ = $d_{2}$. In the following, a typical high-loss material with $\epsilon_{r}^{\prime }$ = 15.0 and tanδ = 0.5 is considered. The sample thickness is 1 mm. Figure 2 shows the errors of $\epsilon_{r}^{\prime }$ and tanδ in each band. It should be emphasized that the errors considered in this paper refer solely to those caused by the air gap. In Figure 2, *d* = $d_{1}$ + $d_{2}$ denotes the total air gap size. It can be seen that the air gap error will increase as the frequency increases. Specifically, for an air gap of 0.04 mm, the maximum error of $\epsilon_{r}^{\prime }$ will increase from 3.4% to 24.4%, and the maximum error of tanδ will increase from 5.6% to 48.1% when the measurement frequency is increased from the X to W band, respectively. In addition, it can be found that both $\epsilon_{r}^{\prime }$ and tanδ errors fluctuate considerably with frequency in the Ka and W bands. When the air gap is 0.04 mm, the errors for $\epsilon_{r}^{\prime }$ can range from −24.4% to −16.8%, while the errors for tanδ can vary from 20.6% to 48.1% between 75 GHz and 110 GHz. Additionally, the error will fluctuate more with frequency as *d* increases. Consequently, the measured trend of $\epsilon_{r}^{\prime }$ and tanδ with frequency may significantly differ from the actual situation. However, the knowledge of the correct trend of dielectric parameter variation with frequency will not only affect the design of dielectric-based devices but is also crucial for studying material dielectric and loss mechanisms. Therefore, the SWM should be improved to meet millimeter-wave measurement requirements.

**Figure 3:**
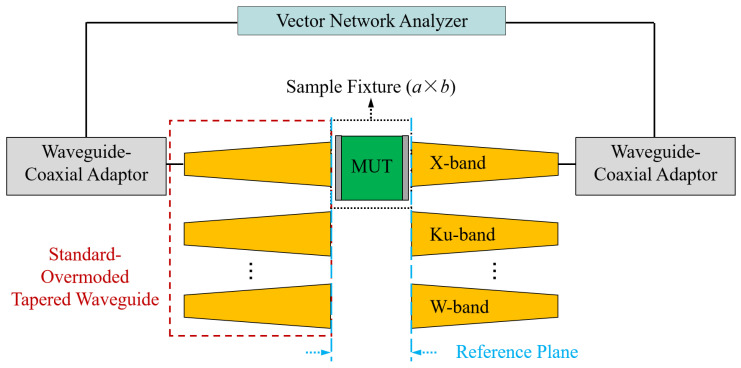
Schematic diagram of the designed OWM-based measurement system.

To achieve a broadband and high-accuracy permittivity measurement from 8 to 110 GHz, we proposed and designed an OWM-based measurement kit. Figure 3 shows the topology of the designed kit. It consists of seven pairs of standard-overmoded-waveguide transition structures (operating on X, Ku, K, Ka, Q, V, and W bands) and an overmoded rectangular waveguide sample fixture with dimensions of 22.86 mm × 10.16 mm. Therefore, only one sample with cross-section dimensions of 22.86 mm × 10.16 mm needs to be fabricated in the 8–110 GHz measurement band. The transition structure converts the standard waveguide ${\mathrm{TE}}_{10}$ mode to the overmoded waveguide ${\mathrm{TE}}_{10}$ mode. The through-reflect-line (TRL) calibration technique will be performed to de-embed the S-matrix of transition structures [19]. For this purpose, we designed the corresponding calibration kits for each band. The length of the “line” components are designed to be 9.8 mm, 5.7 mm, 3.6 mm, 2.4 mm, 2.0 mm, 1.5 mm, and 1.0 mm for each frequency band, respectively, to avoid the phase instability near 0° and 180°. The designed structure parameters are listed in Table 1. It should be noted that although the tapered transition structure is designed to guarantee high-purity transmission of the ${\mathrm{TE}}_{10}$ mode (with mode purity > 99%), the introduced parasitic modes (such as ${\mathrm{TE}}_{30}$, ${\mathrm{TE}}_{50}$, ${\mathrm{TM}}_{12}$, etc.) can still can cause slight resonance and lead to unreasonable extraction results at specific frequencies [13,14]. To address this problem, it is necessary to perform post-processing steps to remove the resonance peaks. In material measurement, time-domain gating technologies or filtering algorithms (used in our work) are widely used, as presented in [20,21].

**Figure 4:**
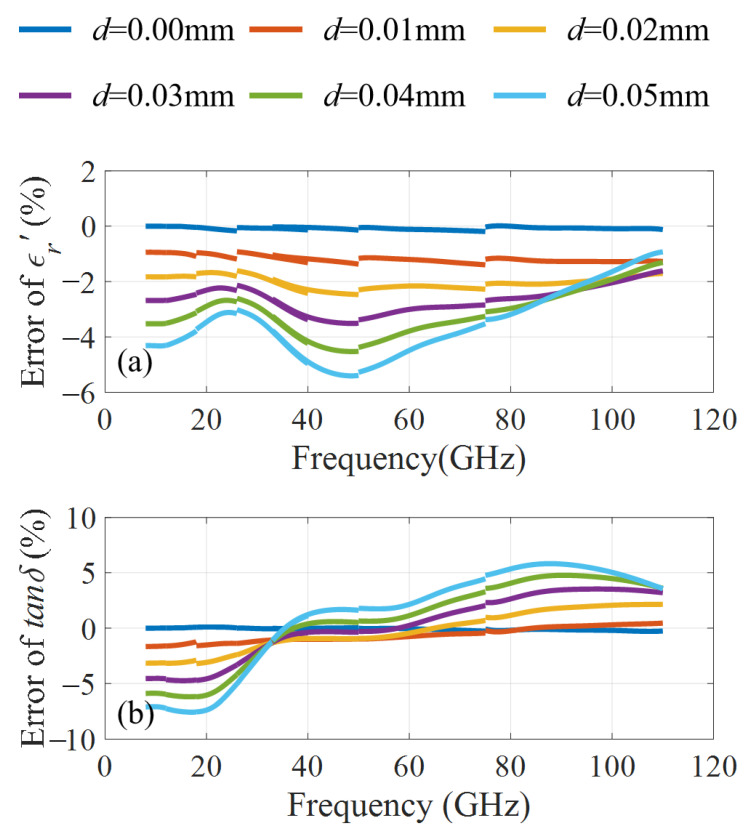
Measurement errors of $\epsilon_{r}^{\prime }$ and tanδ in 8–110 GHz with different air gaps *d*. (**a**) errors of $\epsilon_{r}^{\prime }$; (**b**) errors of tanδ.

Based on the designed kits, we performed an error analysis of the material with $\epsilon_{r}^{\prime }$ = 15 and tanδ = 0.5 in Figure 4. It shows when *d* is smaller than 0.03 mm, the errors of $\epsilon_{r}^{\prime }$ and tanδ does not exceed 5% in 8–110 GHz. When the air gap *d* is increased to 0.05 mm, the errors of $\epsilon_{r}^{\prime }$ and tanδ can be significantly reduced from over 28% and 68% to within 6% and 8%, respectively, compared to SWM. In addition, the fluctuation ranges of $\epsilon_{r}^{\prime }$ and tanδ errors will be reduced from 24% and 75% to 5% and 13%, respectively, over the whole measurement band from 8 to 110 GHz. These results indicate that the designed OWM-based measurement kit is much more accurate than SWM and does not require sample replacement during measurement in different frequency bands. These excellent properties give the designed kit potential for a wide range of applications.

## 3. Measurement Results

**Figure 5:**
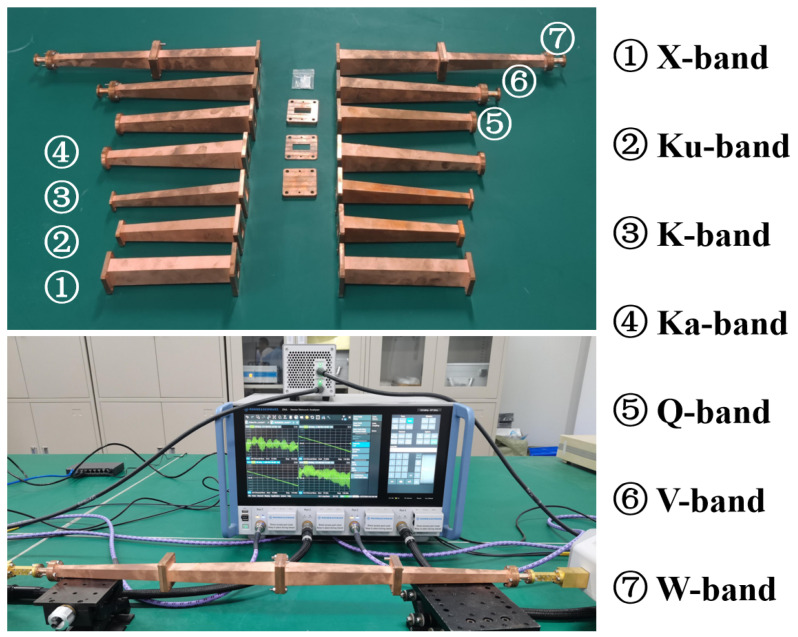
VNA measurement platform and the fabricated OWM-based kit.

The experimental verification and application of the proposed measurement kit will be presented in this section. Figure 5 shows the vector network analyzer (VNA) measurement platform and the fabricated OWM-based kit. Although the waveguide method is more suitable for measuring high-loss materials, there is a lack of reported standardized data for the complex permittivity of high-loss materials to assess measurement accuracy. In order to verify the designed kit, we measured the well-known low-loss polytetrafluoroethylene (PTFE) and compared the measured results with reported data obtained by various broadband measurements. Furthermore, the OWM-based kit is applied to a high-loss BeO-TiO_2_ ceramic measurement.

### 3.1. Measurement Verification of the PTFE

**Figure 6:**
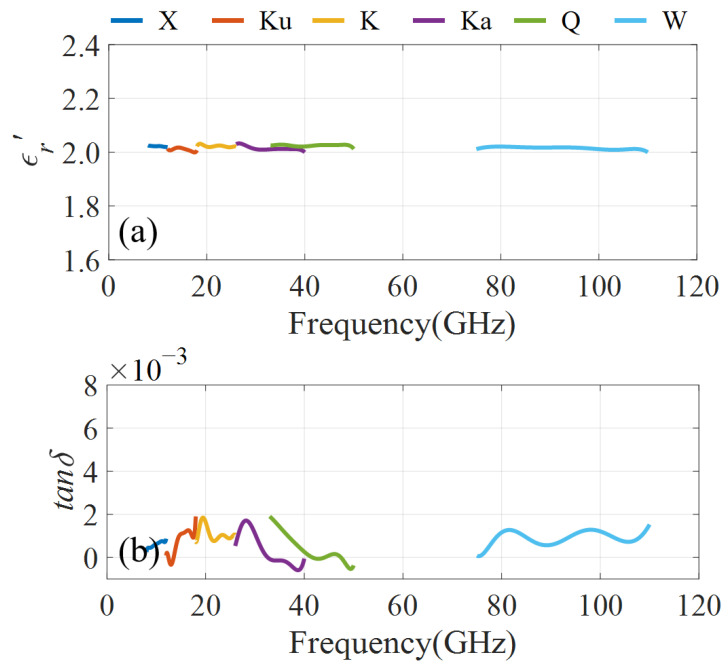
Measurement results of the PTFE. (**a**) $\epsilon_{r}^{\prime }$, (**b**) tanδ.

In our measurement, the PTFE sample has a thickness of 1 mm. The measurement results are shown in Figure 6. It should be mentioned that due to the limitations of our laboratory equipment, we did not measure V-band (50–75 GHz). Nevertheless, since the measurement band is sufficiently wide, it does not affect our conclusion. From Figure 6 we can find that the average $\epsilon_{r}^{\prime }$ and tanδ is 2.02 and 0.001. Since the waveguide method does not yield an accurate tanδ for low-loss materials (tanδ < 0.001), we just compare the extracted $\epsilon_{r}^{\prime }$ with the reported data. The measurement results and reported data are summarized in Table 2. It can be seen that our measurement results agree well with the reported data in the 8–110 GHz range, which effectively verifies the feasibility of the OWM-based kit.

### 3.2. Application in Lossy Material Measurement

A high-loss BeO-TiO_2_ ceramic is measured using our designed measurement kit. It is produced of BeO ceramic with TiO_2_ powder [27]. The complex permittivity of the BeO-TiO_2_ ceramics can be adjusted by changing the proportion of BeO and TiO_2_. The $\epsilon_{r}^{\prime }$ and tanδ of BeO-TiO_2_ ceramics are typically in the range of 5–30 and 0.1–5. Due to its high-loss property, BeO-TiO_2_ ceramics are can be used in absorbers [28], power beam applications [2], and vacuum devices [29]. Since this material is susceptible to composition and sintering processes, it is necessary not to replace samples during broadband measurement in different frequency bands.

**Figure 7:**
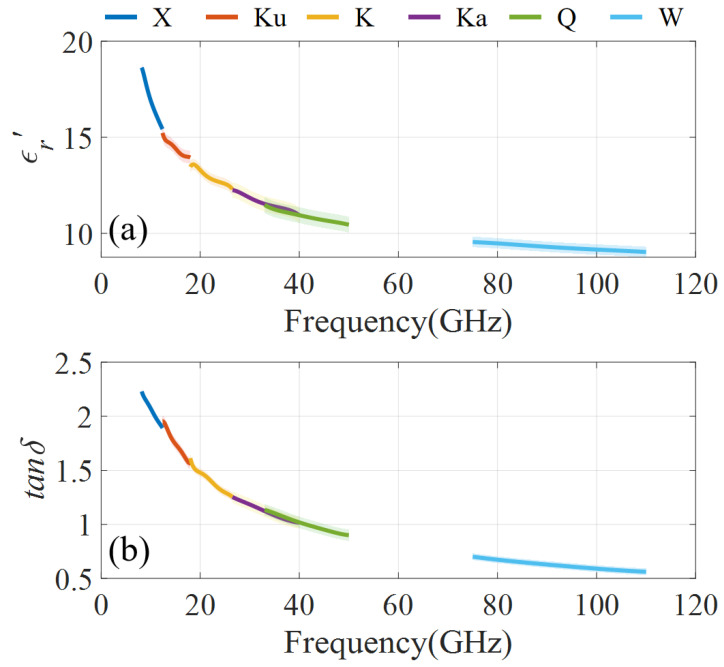
Measurement results of the BeO-TiO_2_ ceramic. (**a**) $\epsilon_{r}^{\prime }$, (**b**) tanδ.

Figure 7 shows the measured $\epsilon_{r}^{\prime }$ and tanδ of the BeO-TiO_2_ ceramic. The BeO-TiO_2_ sample under measurement has a thickness of 1 mm. In Figure 7, the shaded area shows the uncertainties caused by VNA noise and repeated measurements. They are ±0.4 and ±0.005 for $\epsilon_{r}^{\prime }$ and tanδ, respectively. It can be found that both the $\epsilon_{r}^{\prime }$ and tanδ show a decreasing trend with the frequency. They decrease from 18.63 to 9.03 and 2.23 to 0.56 in 8–110 GHz, respectively. Since the measurement results in different frequency bands are consistent, these results can be considered credible. These data provide essential information for developing high-loss BeO-TiO_2_ ceramics and its application.

## 4. Conclusions

This paper presents an ultra-broadband OWM-based measurement kit operating in 8–110 GHz with high accuracy. Simulation analysis indicates the errors of $\epsilon_{r}^{\prime }$ and tanδ can be controlled within 6% and 8% with a large E-plane air gap of 0.05 mm for measurements of high-loss material. The PTFE sample is measured and compared with the published references to verify the designed kit. Then, it is applied in the measurement for high-loss BeO-TiO_2_ ceramic. Based on the simulation and measurement results, the proposed method is an effective solution for measuring ultra-broadband properties of the complex permittivity, which contributes to the study of dielectric mechanisms and the application of materials. 

## Figures and Tables

**Table 1:** Structure parameters of the OWM-based measurement kits.

Frequency Band	X	Ku	K	Ka	Q	V	W
Frequency Range (GHz)	8–12	12–18	18–26	26–40	33–50	50–75	75–110
Overmoded Factor $g_{a}$	1	1.45	2.14	3.21	4.02	6.08	9.00
Overmoded Factor $g_{b}$	1	1.29	1.90	2.86	3.57	5.41	8.00
Taper length (mm)	140	140	140	160	180	250	300
Line length (mm)	9.8	5.7	3.6	2.4	2.0	1.5	1.0
**Sample Fixture Size (mm)**	**22.86 × 10.16 mm**						

**Table 2:** Reported permittivity of the PTFE.

Reference	Measurement Frequency	$\epsilon_{r}^{\prime }$	Measurement Method
[22]	1–18 GHz	2.03–2.04	Coaxial Line
[23]	26–40 GHz	1.95	Dielectric Waveguide
[24]	50–75 GHz	1.99–2.01	Guided Free-space
[25]	80–105 GHz	1.99–2.00	Circular Waveguide
[26]	75–110 GHz	2.05	Free-space Method
This work	8–110 GHz	2.00–2.03	OWM

## Data Availability

All data are included in the manuscript.
